# Supplementary figures and images for: Amphotericin B resistance in Leishmania mexicana: Alterations to sterol metabolism and oxidative stress response
Source: PLoS Negl Trop Dis. 2022 Sep 28;16(9):e0010779. doi: 10.1371/journal.pntd.0010779 (PMC9581426; doi:10.1371/journal.pntd.0010779)

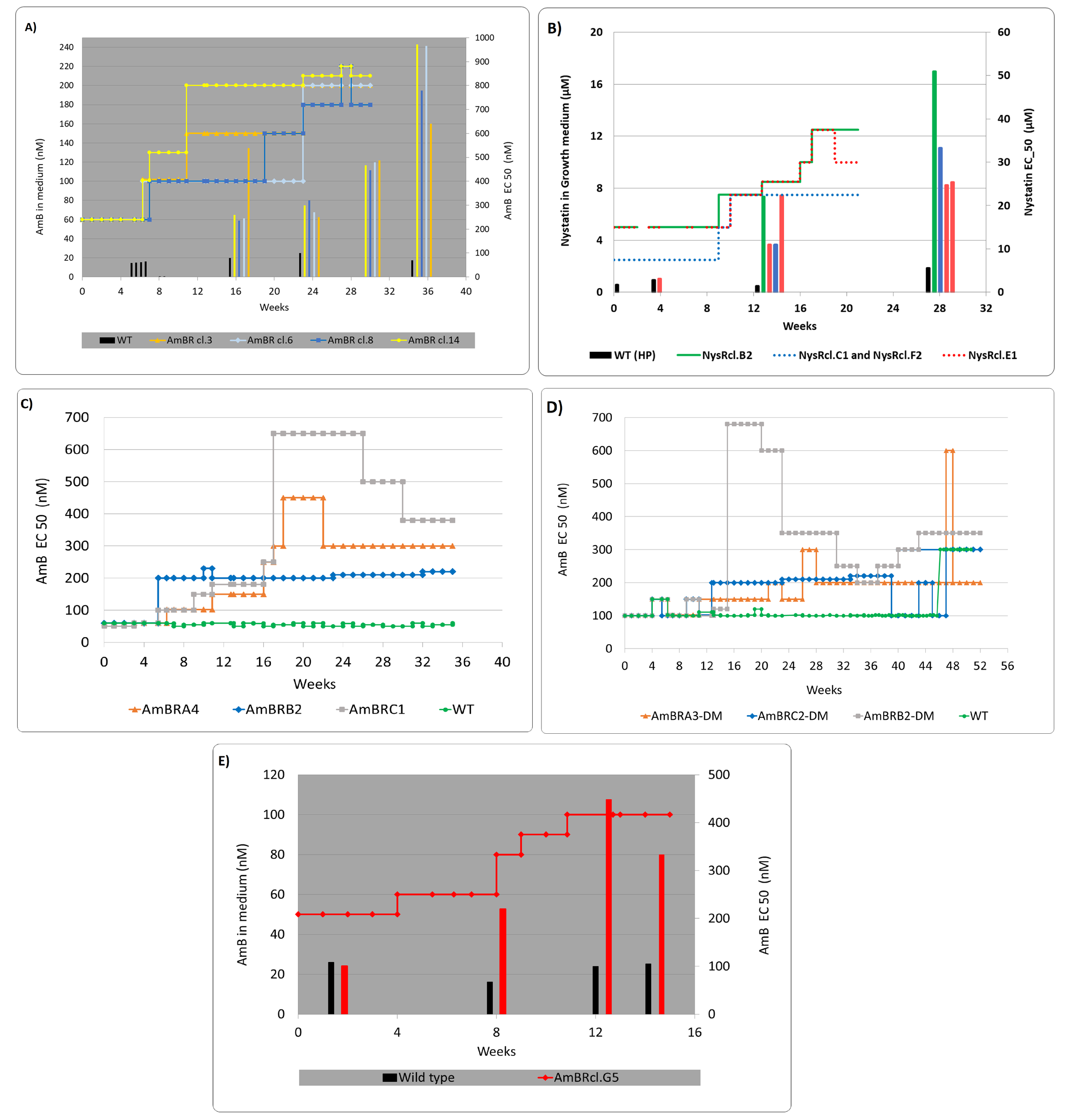

Supplement: S1 Fig — L. mexicana (S1A to S1D Fig) and L. infanum (S1E Fig) mid-log promastigotes (5x10^5^) were growth with a stepwise increasing concentration of AmB (S1A, S1C-E Fig) or Nys (S1B Fig) added in the cultured medium and indicated with coloured pipelines (left y-axis). The EC50 (right-y axis) is shown for wild type (black bars) and resistant lines, AmBR and NysR (coloured bars in all panels). The mean EC50 of the parental wild type is shown (horizontal dotted lines). See Material and Methods for a detailed description. (TIF) [file pntd.0010779.s010.tif]

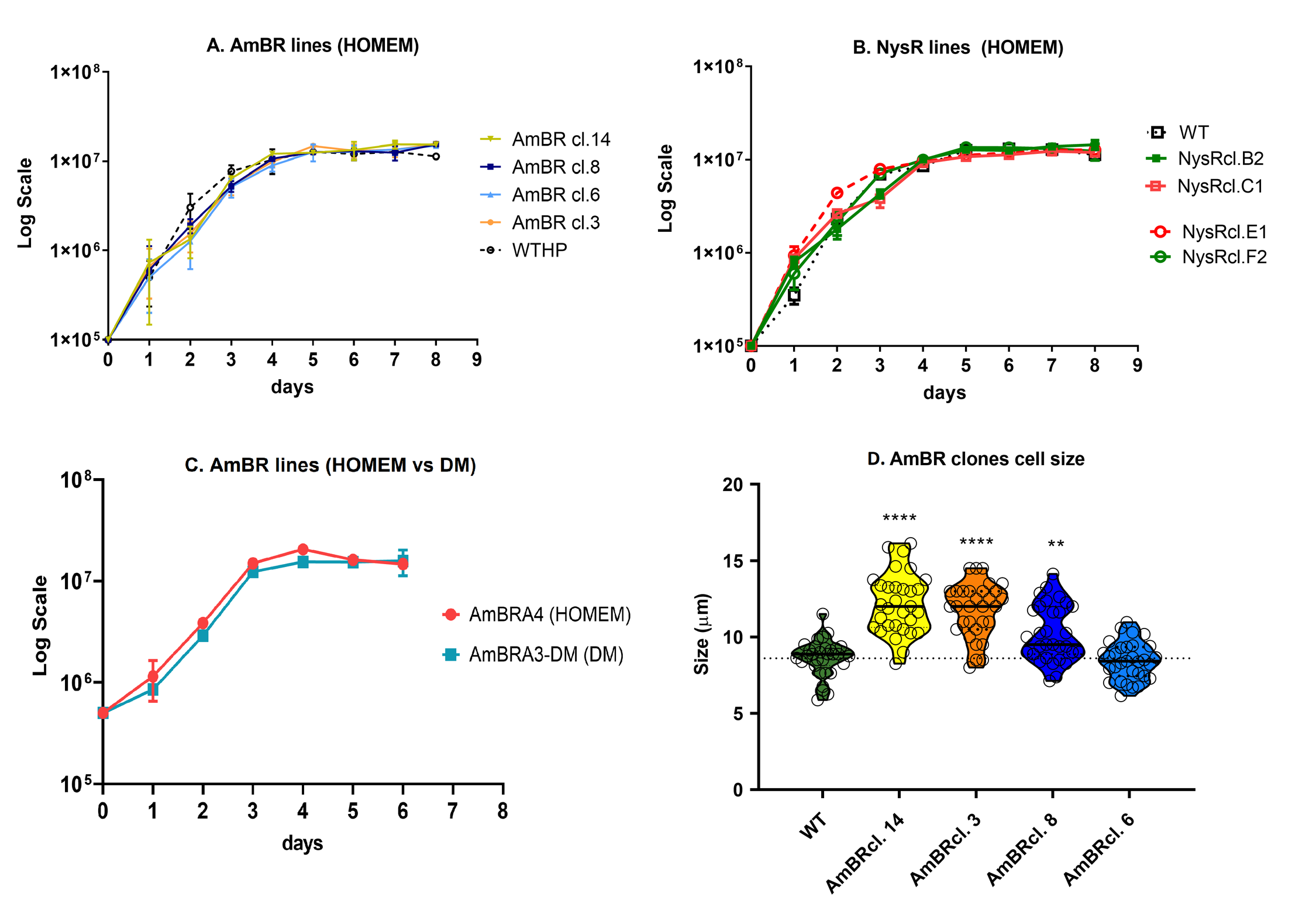

Supplement: S2 Fig — Cell density was measured every 24 hours for 8 days starting from 1x10e5 cells/ml. S2A Fig) AmBR lines in HOMEM. S2B Fig) NysR in HOMEM. S2C Fig) AmBRA4 in HOMEM and AmBRA3-DM in Defined Medium (DM). S2D Fig) Violin plot of the mean cell body length (μm) of four AmBR lines from panel A showing AmBRcl.14 and AmBRcl.3 with an increased length relative to wild type. The central continuous line within each coloured violin plot is the median of each group. The cell body length was measured from the base of the flagellum until the posterior endpoint of the cell body of promastigotes in the stationary phase. Data were processed with ImageJ software and represent the mean of the sample (n≥ 30). Measurements are the mean (±SD) of three biological replicates. Tukey’s multiple comparison test was used to find pairwise differences between resistant lines and parental wild type. Statistically significant values (P<0.05, 95% Confidence Interval) are indicated with stars as follows: *P ≤ 0.05, **P ≤ 0.01, ***P ≤ 0.001, ****P ≤ 0.0001). (TIF) [file pntd.0010779.s011.tif]

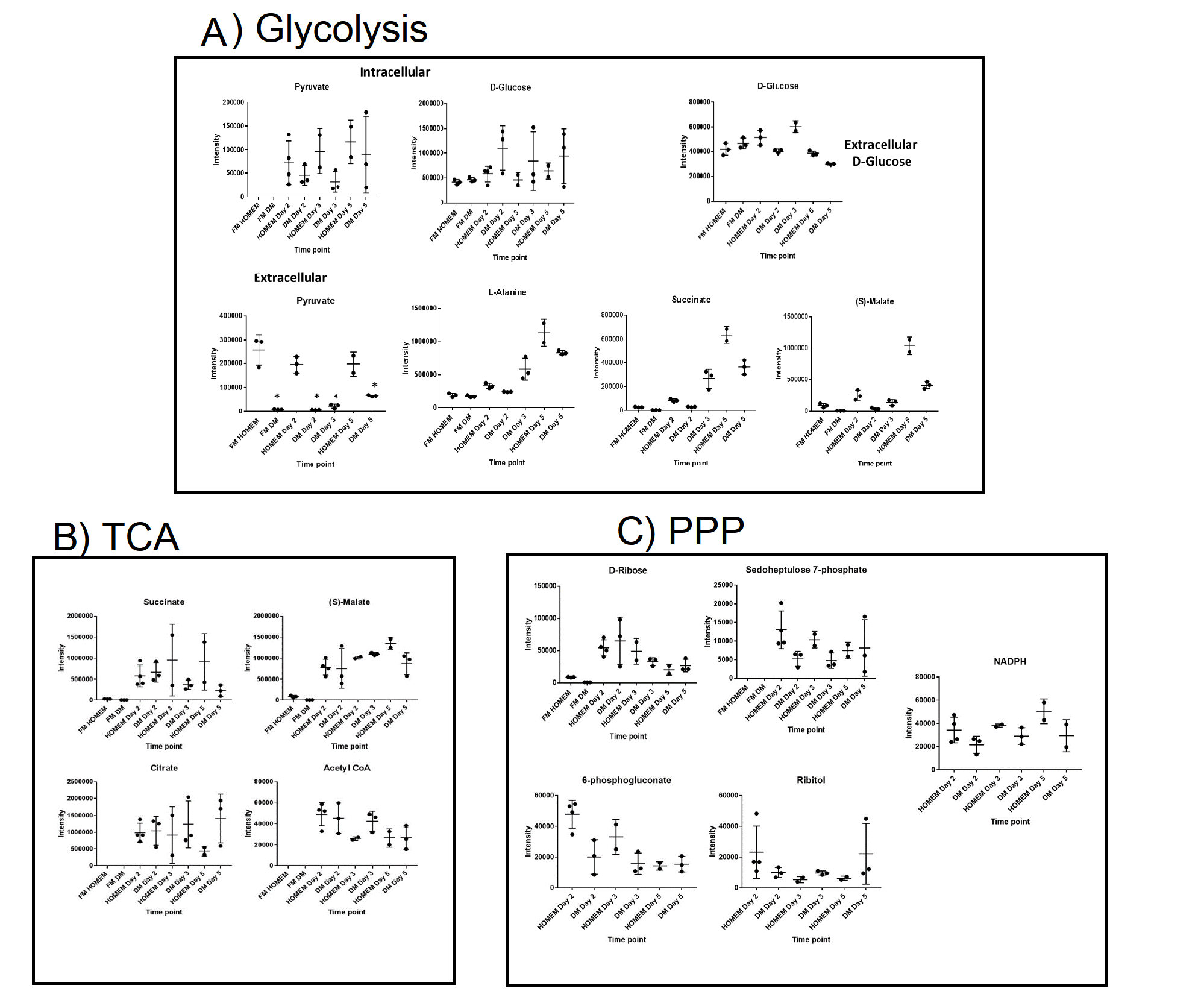

Supplement: S3 Fig — Metabolites in the glycolysis (S3A Fig), citric acid cycle (TCA) (S3B Fig) and Pentose Phosphate Pathway (PPP) (S3C Fig) detected with LC-MS in mid log wild type parasites (1 x 10e8) cultured in HOMEM or DM. Multivariate data analysis was performed with PiMP analysis pipeline [115] and the Benjamini-Hochberg procedure adjusted raw P-values (q-values) < 0.05 for ANOVA. Each experiment represents one biological replicate (n = 4). (TIF) [file pntd.0010779.s012.tif]

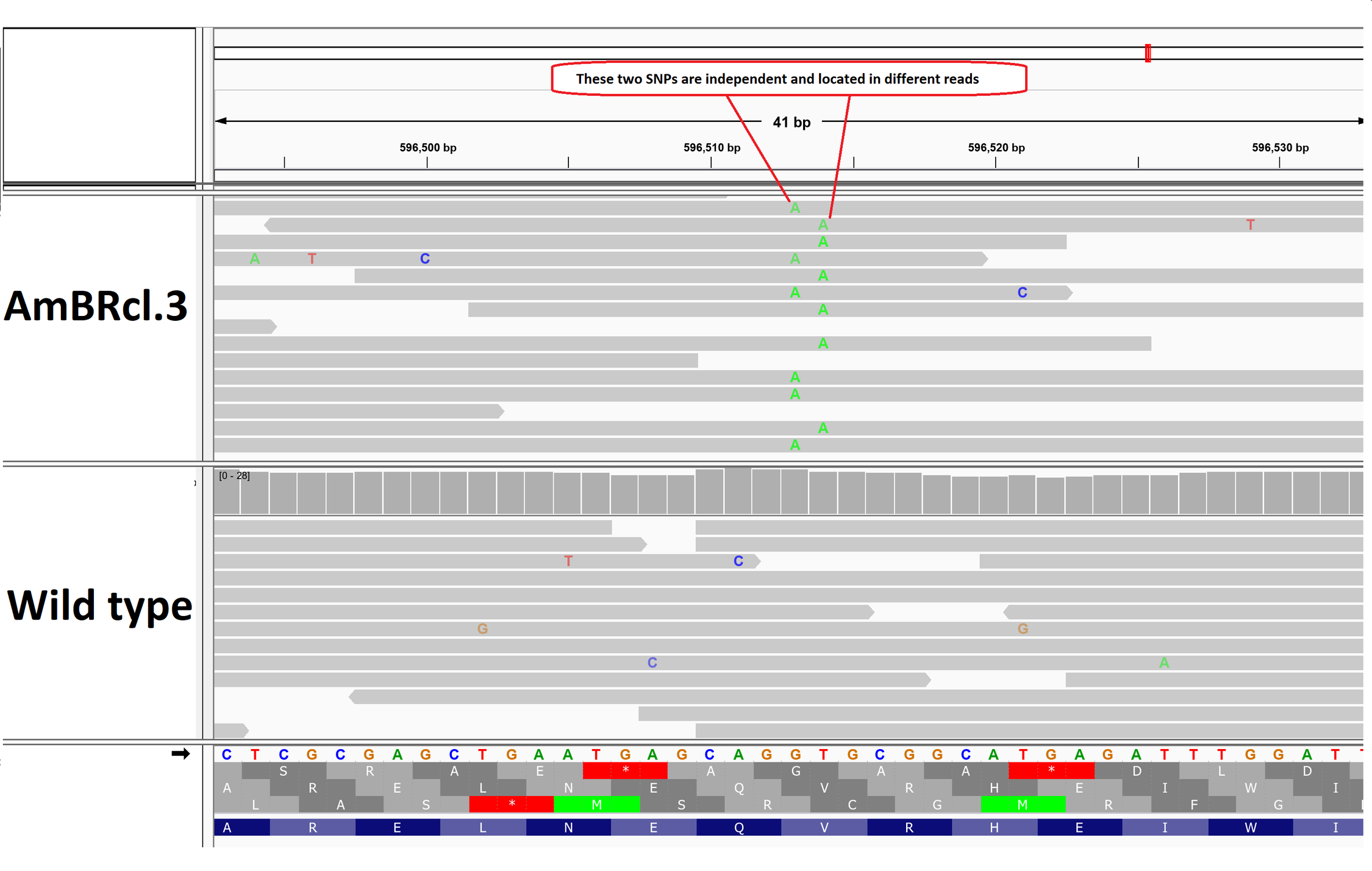

Supplement: S4 Fig — The image shows two independent heterozygous mutations, G220A (V74M) and T221A (V74E) in AmBRcl.3. The red square indicates the localisation of both mutation in different reads. Image was produced with WGS data aligned to the reference genome (https://tritrypdb.org) using the IGV_2.8.9 software (http://software.broadinstitute.org/software/igv/). See Fig 5 for a full description of the panels. (TIF) [file pntd.0010779.s013.tif]

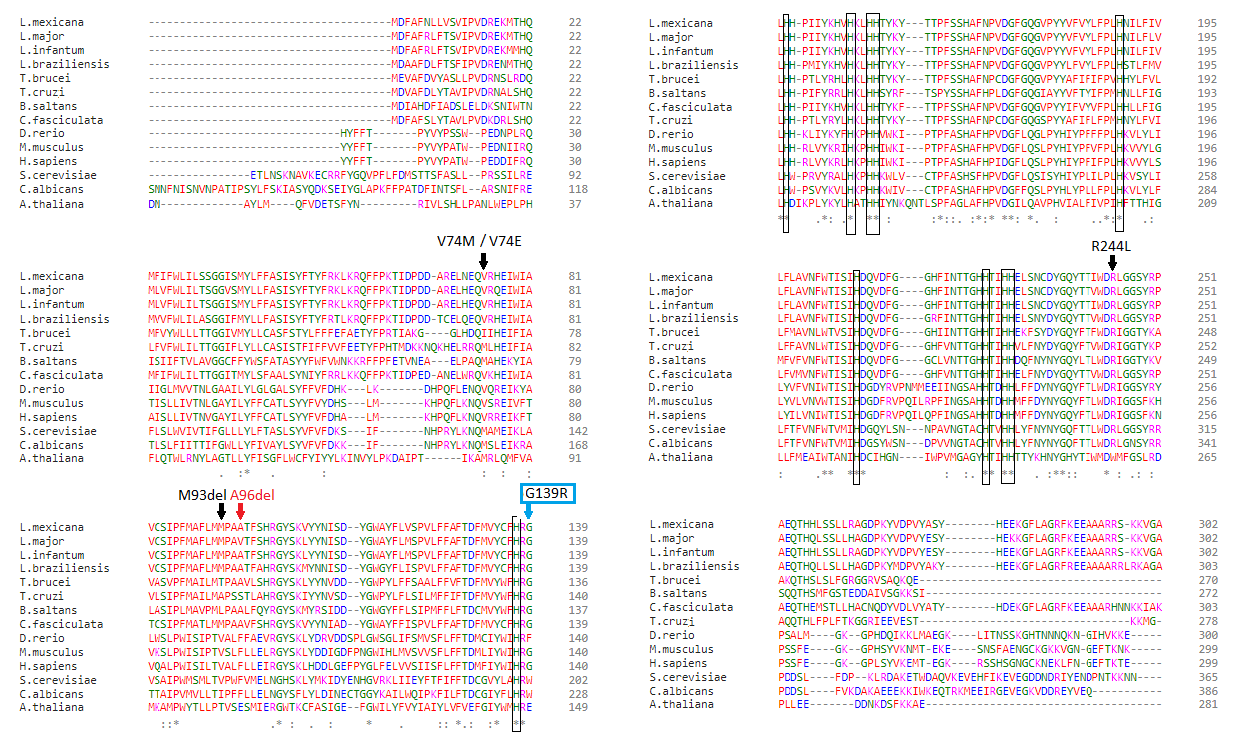

Supplement: S5 Fig — L. mexicana gene LmxM.23.1300 (LOX). Black boxes and stars denote His and other residues conserved across species, respectively. Novel variants identified in this study are indicated with black (AmBR lines) and red (NysR line) arrows. Also included (blue box) is the variant from our previous study [96]. From top to bottom kinetoplastids listed are L. mexicana, L. major, L. infantum, L. braziliensis, T. brucei, T. cruzi, Bodo saltans and Crithidia fasciculata. Also included are D. rerio (zebra fish), M. musculus (mouse), Homo sapiens (human), S. cerevisiae (budding yeast), C. albicans (pathogenic fungi) and Arabidopsis thaliana (plant). Proteins sequences are from TriTrypDB (https://tritrypdb.org) and Uniprot (https://www.uniprot.org/). Alignment was performed using Clustal Ω with default settings (https://www.ebi.ac.uk/Tools/msa/clustalo/). (TIF) [file pntd.0010779.s014.tif]

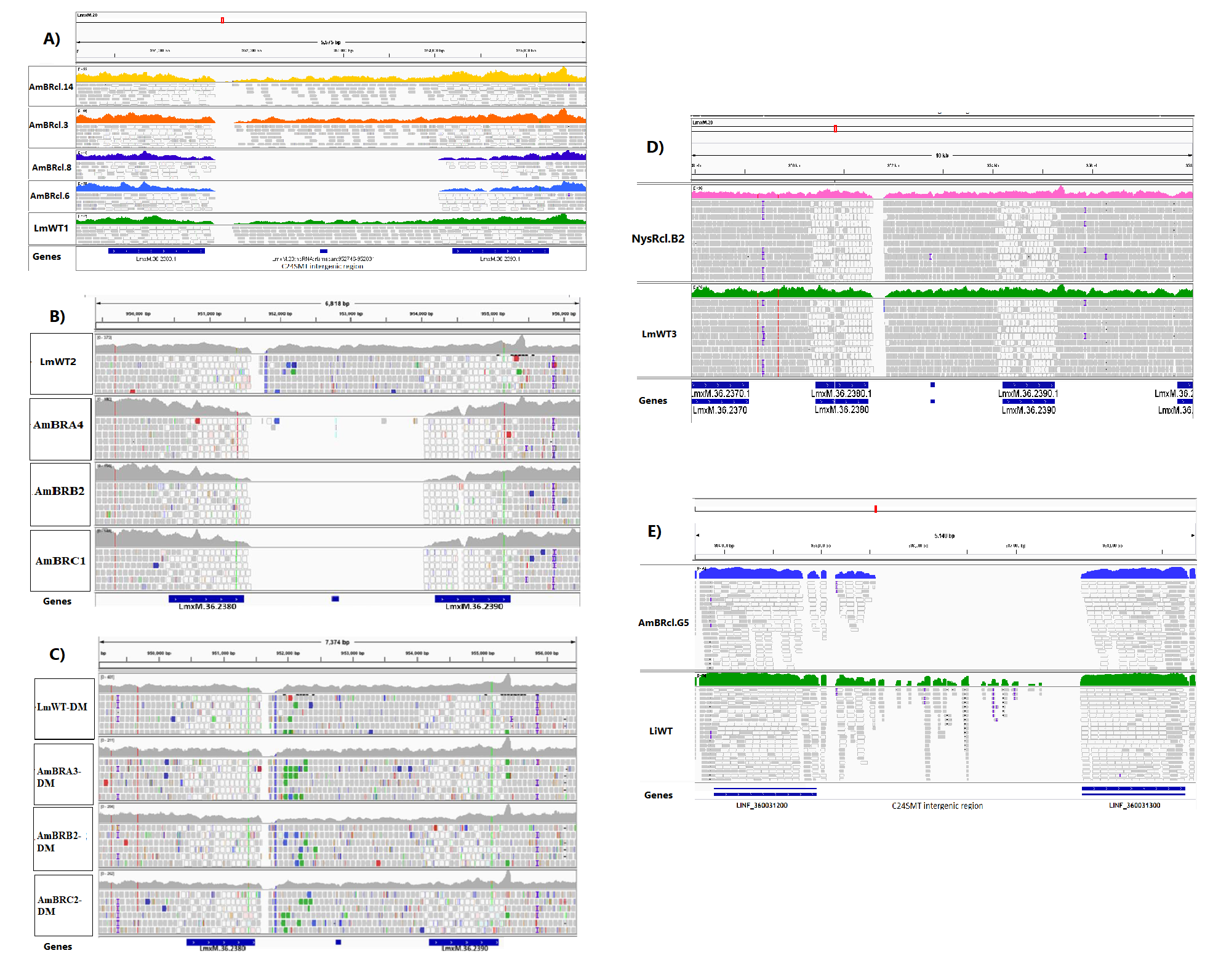

Supplement: S6 Fig — S6A-B Fig) AmBR lines (and their respective parental WT) cultured in HOMEM showing the absence of coverage in this locus in five lines. S6C Fig) AmBR lines and wild type cultured in DM. S6D Fig) Reads coverage in the intergenic region in NysRcl.B2 and its parental WT. S6E Fig) L. infantum showing total absence (AmBRcl.G5) and partial coverage (parental WT). Reads were aligned to the reference genome (https://tritrypdb.org) and the image was produced using the software IGV 2.8.9 (http://software.broadinstitute.org/software/igv/). See Fig 5 for a full description of the panels. (TIF) [file pntd.0010779.s015.tif]

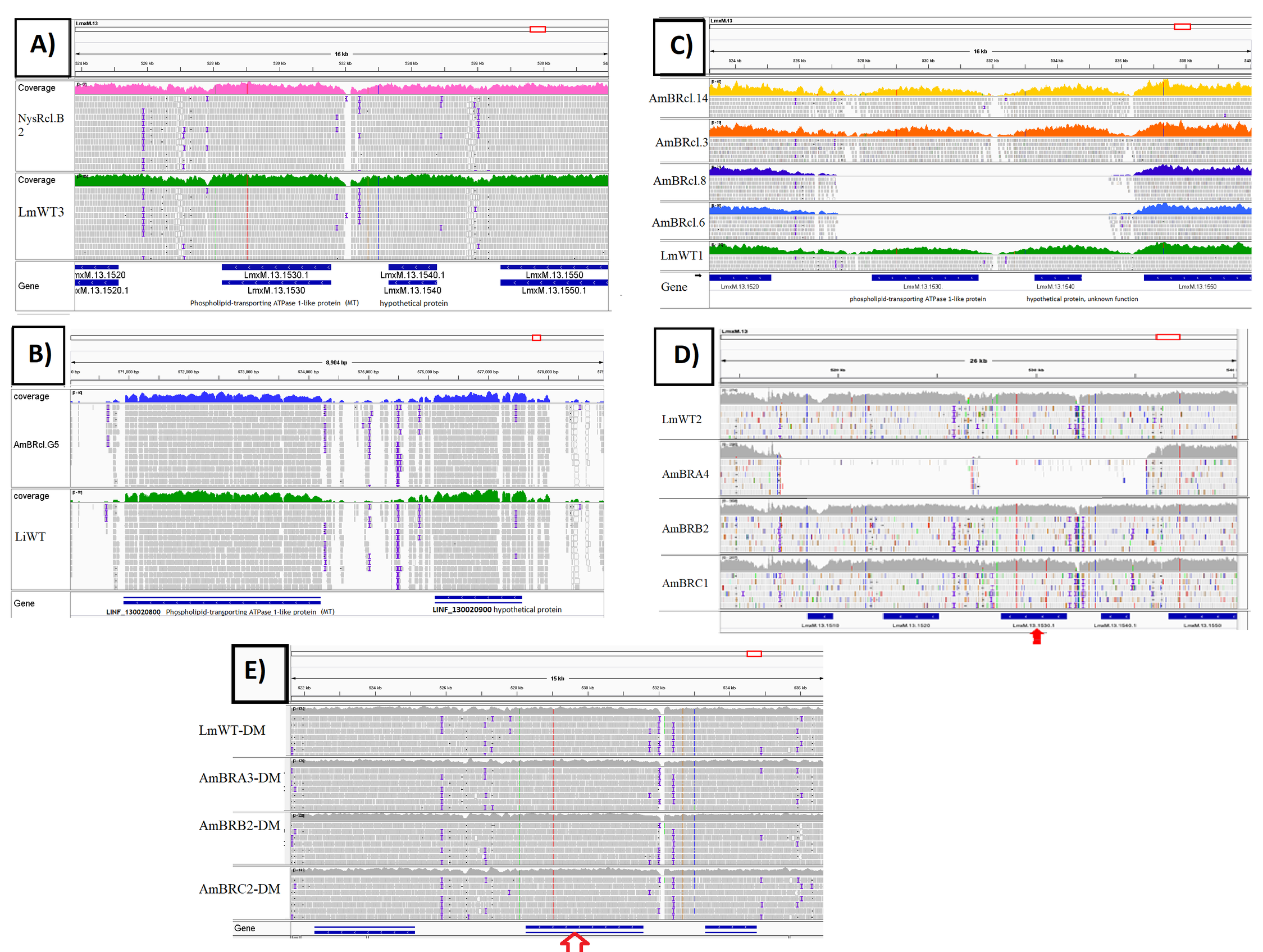

Supplement: S7 Fig — The coordinates of a region spanning a genomic region of ~16 kb and ~26 kb are shown. Genes are shown at the bottom (blue bars). S7A-B Fig) coverage in NysRcl.B2 and AmBRcl.G5 S7C Fig) In AmBRcl.8 and AmBRcl.6 a total absence of coverage of a region (~9 kb) comprising LmxM.13.1530 and adjacent gene downstream LmxM.13.1540 is shown while small gaps of low coverage are observed downstream from each gene in AmBcl.14, AmBcl.3 and WT. S7D Fig) Absence of a ~20 kb region comprising four genes (from LmxM.13.1510 to LmxM.13.1540) is shown in AmBRA4. WGS data were aligned to the reference genome (https://tritrypdb.org) and the images were produced with IGV_2.8.9 software (http://software.broadinstitute.org/software/igv/). See Fig 5 for a full description of the panels. (TIF) [file pntd.0010779.s016.tif]

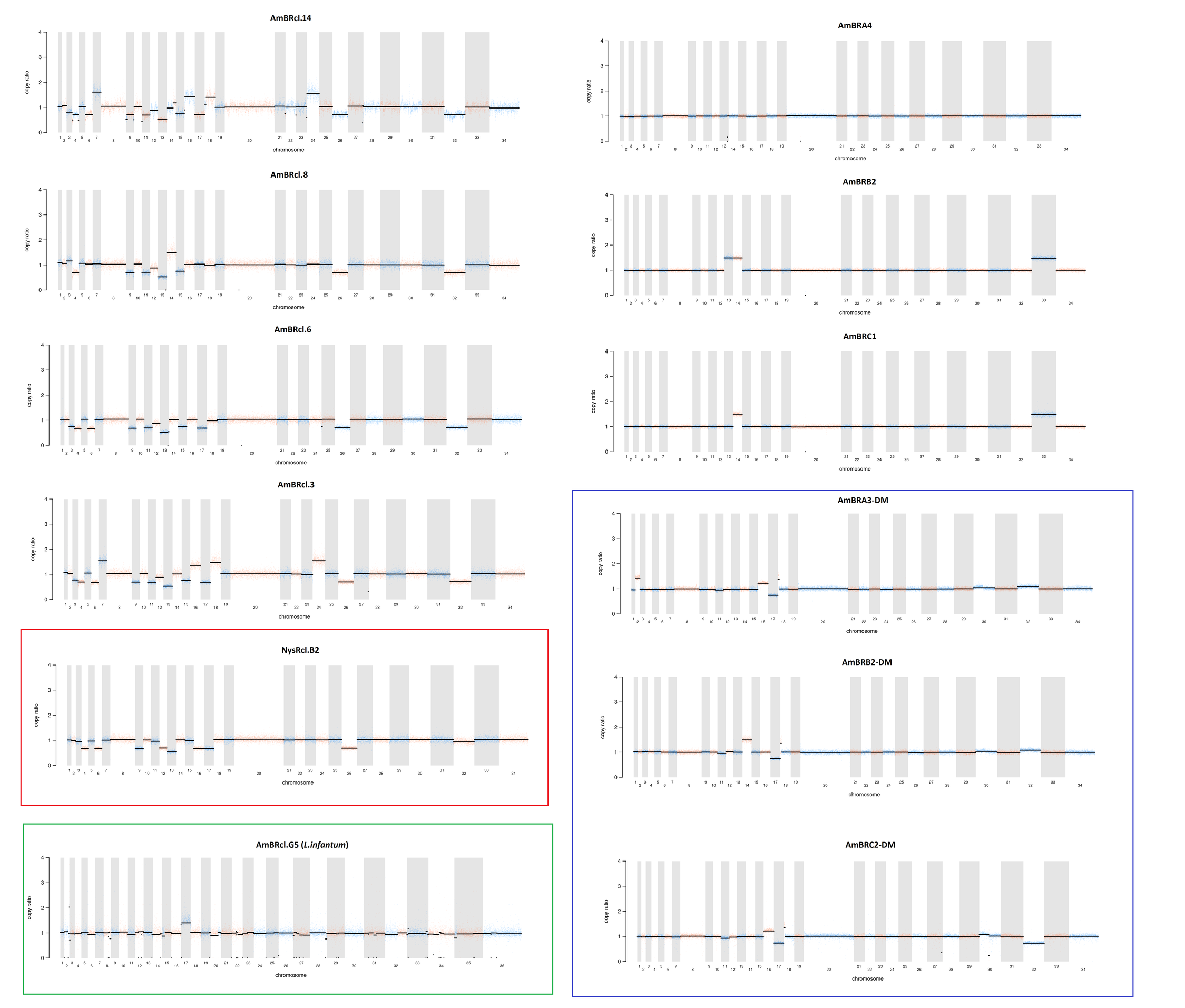

Supplement: S8 Fig — Copy ratio alterations were detected using GATK (version 4.2.0.0). Reference genomes from TriTrypDB46 (https://tritrypdb.org) were divided into equally sized bins of 1000 base pairs (start and end points of the called segments were approximate). CN ratio is obtained by comparing the resistant lines with the corresponding wild type line. Plots of denoised and segmented copy-ratios were generated using R. The thick black lines show the mean copy ratios of corresponding segments. Values of this Fig are provided in S8 Table. (TIF) [file pntd.0010779.s017.tif]

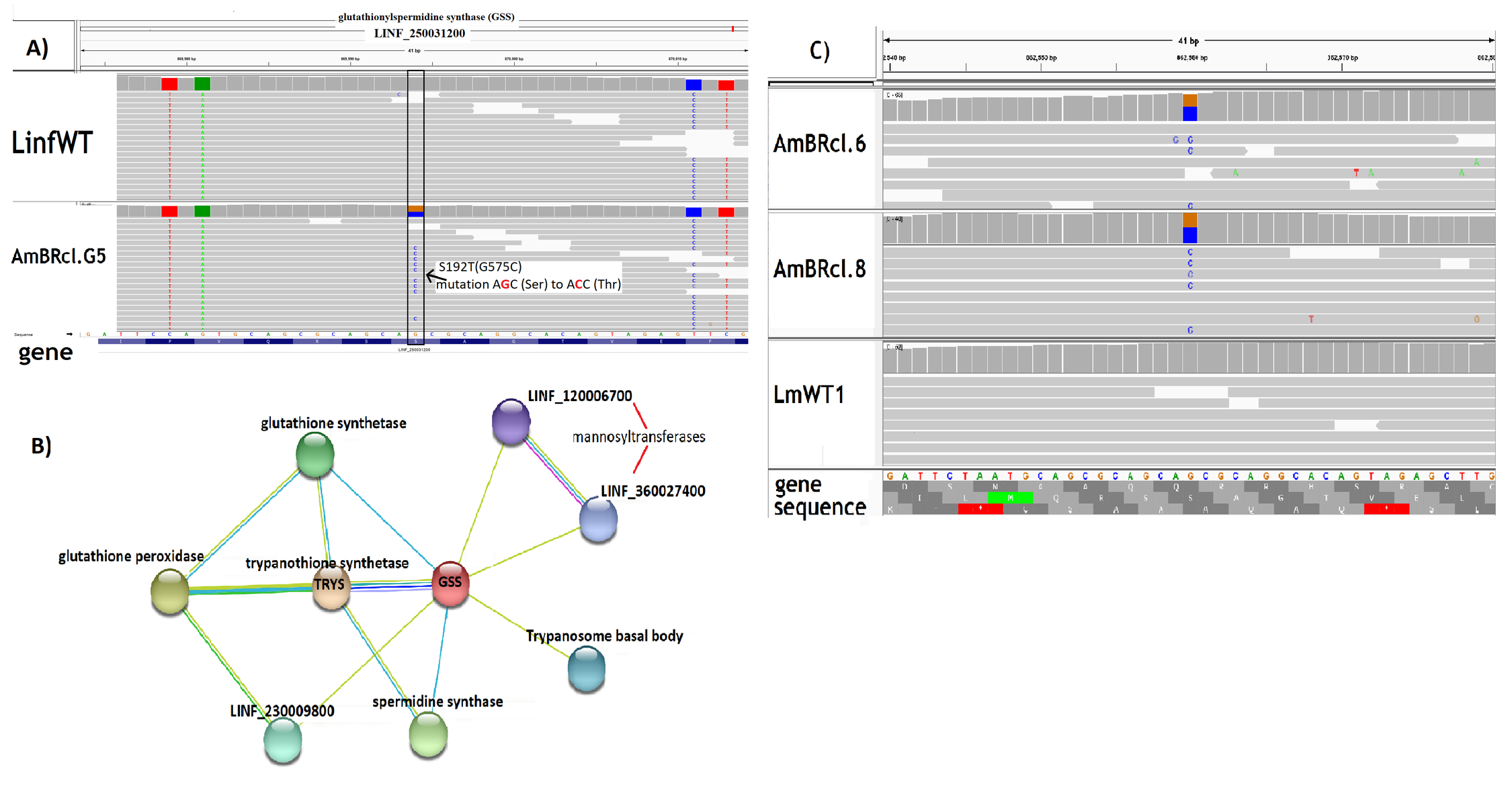

Supplement: S9 Fig — S9A Fig) Genomic region showing a heterozygous mutation (boxed) in GSS in AmBRcl.G5 of L. infantum. S9B Fig) Protein-protein interaction (PPIs) map of GSS and other enzymes relevant for the trypanothione biosynthesis. GSS (LINF_250031200), GSS: glutathione synthetase (LINF_140015200), TRYS: trypanothione synthetase (LINF_270025600), glutathione peroxidase (LINF_360038100), trypanothione synthetase (LINF_230009800), spermidine synthase (LINF_040010800), trypanosome basal body component (LINF_240028000) and two mannosyltransferases (LINF_360027400 and LINF_120006700). S9C Fig) Genomic region showing the heterozygous mutation in GSS in two lines, AmBRcl.8 and AmBRcl.6 of L. mexicana. The reference genome and gene IDs were retrieved from TriTrypDB (https://tritrypdb.org). Images were produced using IGV 2.8.9 (http://software.broadinstitute.org).and STRING (https://string-db.org). See Fig 5 for a full description of the S9A and S9C Fig. (TIF) [file pntd.0010779.s018.tif]

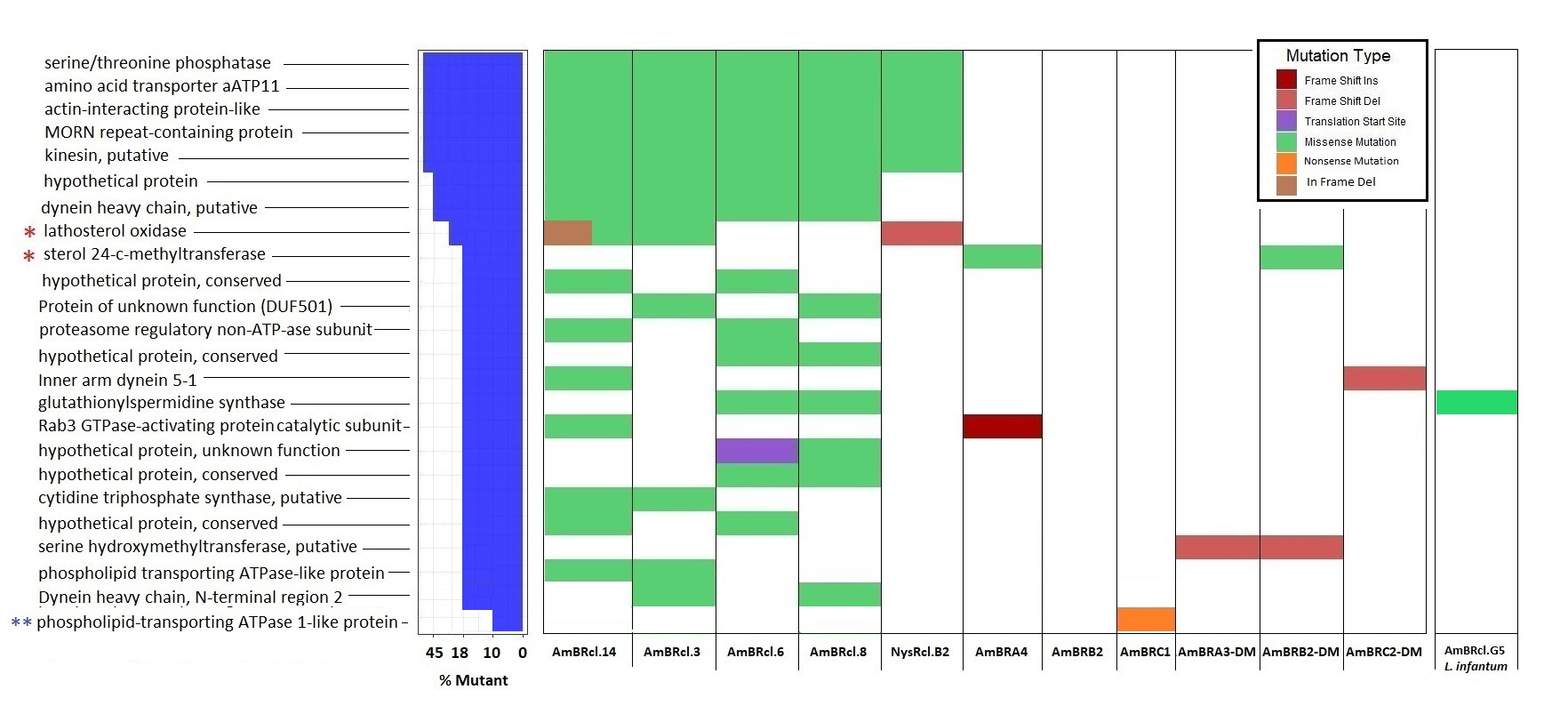

Supplement: S10 Fig — (List of genes). Gene ID shown on the left were retrieved from TriTrypDB. All polyene resistant lines are shown in the x-axis and the mutation(s) type present in each are coloured coded. This image was produced with R package. See S9 Table for a full list of these variants. (TIF) [file pntd.0010779.s019.tif]

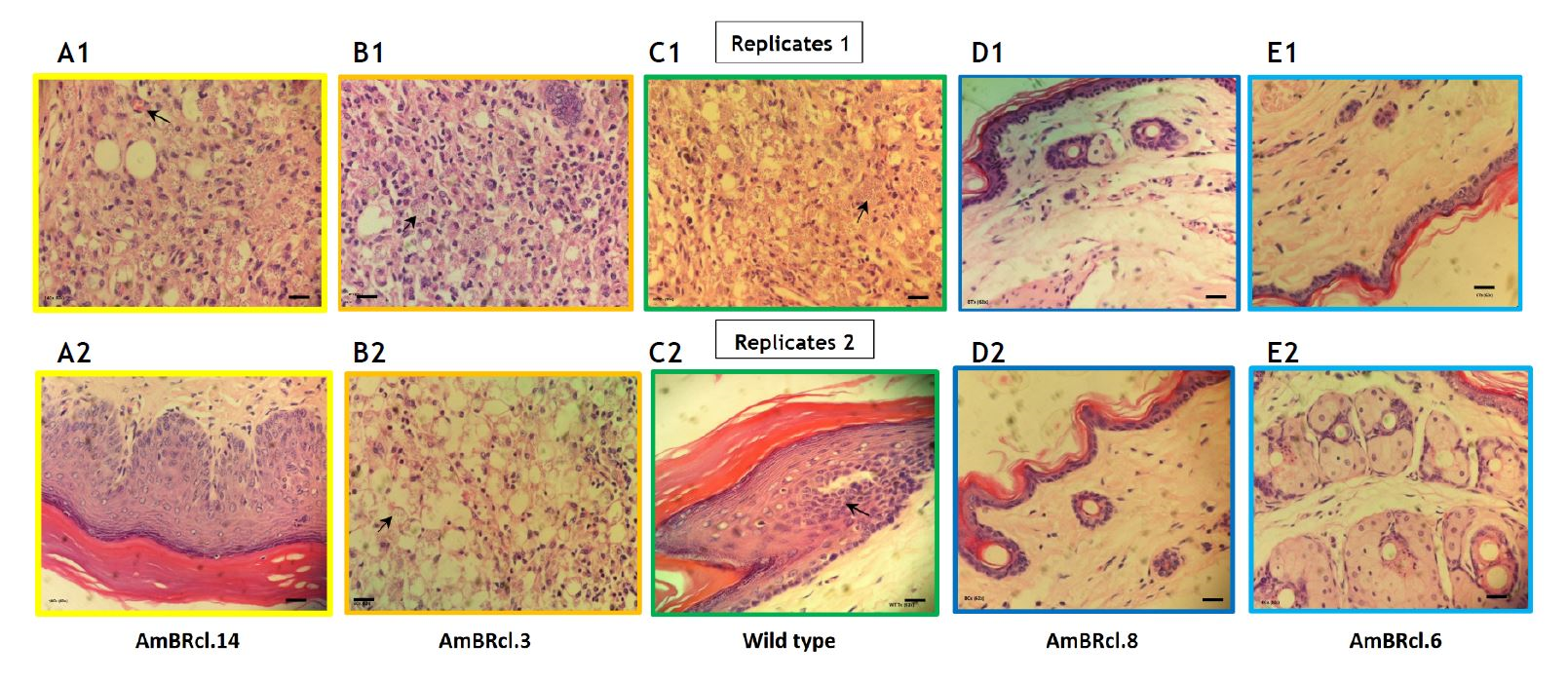

Supplement: S11 Fig — S11A1-A2, S11B1-B2 Fig) AmBRcl.14 (yellow) and AmBRcl.3 (orange) infection with intense parasitic load and inflammatory infiltrate within skin histiocytes. S11C1-C2 Fig) L. mexicana wild type (green) causing diffuse inflammatory infiltration. Internalised amastigotes are indicated (black arrows). S11D1-D2, S11E1-E2 Fig) infection with AmBRcl.8 and AmBRcl.6 (dark and light blue) and discrete inflammatory reaction localised in papillary dermis without visible parasites. Samples are representative of three biological replicates. Hematoxylin-eosin (H&E) stain. Scale bar ~10 μM Objective ~63x. (TIF) [file pntd.0010779.s020.tif]

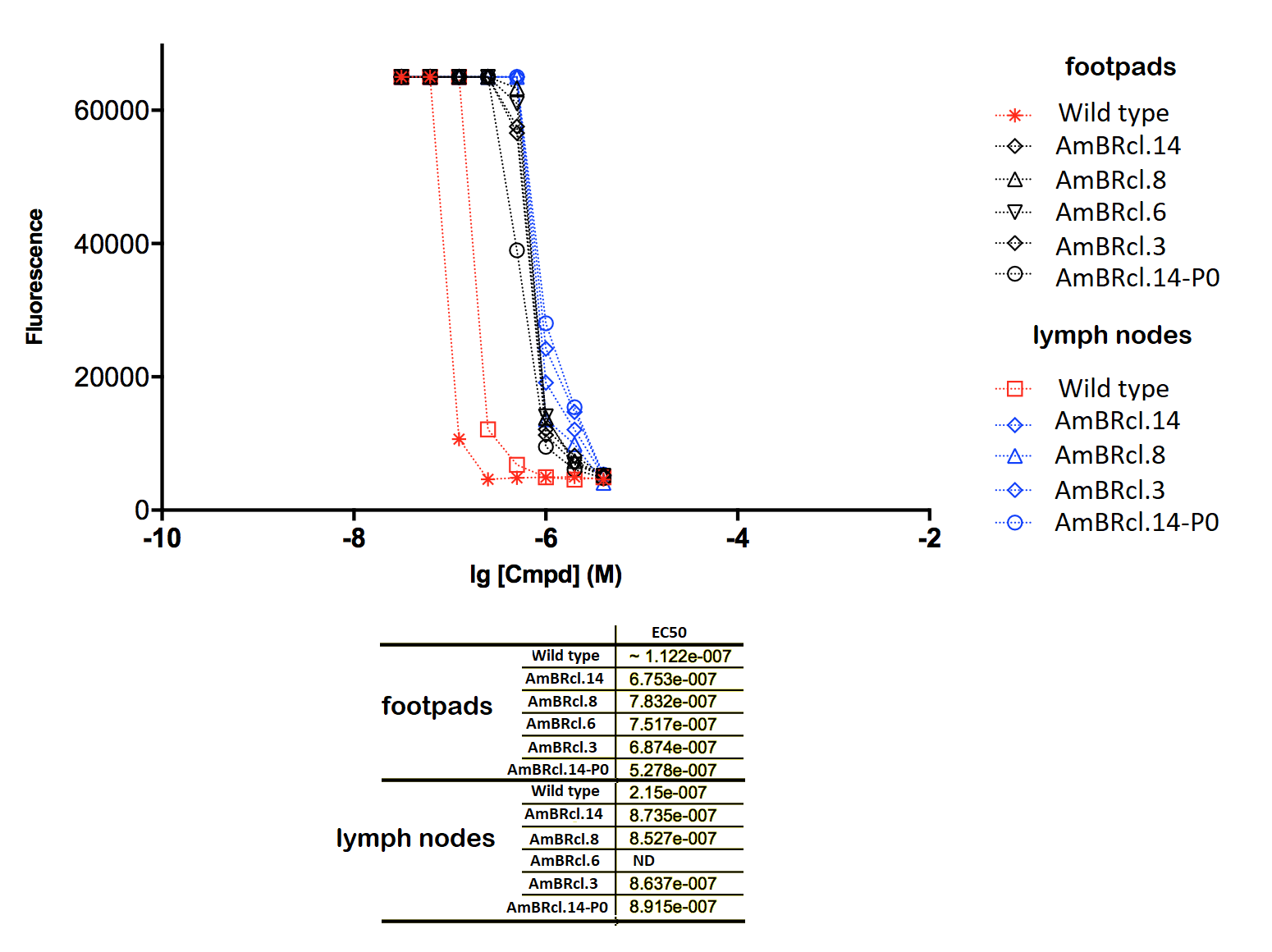

Supplement: S12 Fig — Susceptibility to AmB in AmBR L. mexicana axenic promastigotes after infection in mice. Parasites were recovered as amastigotes from mice tissue (lymph nodes and footpad) and transformed into promastigotes in HOMEM culture medium. Mice were treated with AmB (1 mg/kg) at week thirteen or left untreated (control group). See Material and Methods for a full description. (TIF) [file pntd.0010779.s021.tif]
