# Supplementary material for: Amphotericin B resistance in Leishmania mexicana: Alterations to sterol metabolism and oxidative stress response
Source: PLoS Negl Trop Dis. 2022 Sep 28;16(9):e0010779. doi: 10.1371/journal.pntd.0010779 (PMC9581426; doi:10.1371/journal.pntd.0010779)
Supplement: Attachment — Submitted filename: Comments AmB resis plost ntd 22.docx [file pntd.0010779.s022.docx]

Comments:

The manuscript entitled “ Amphotericin resistance in L. Mexicana : alteration to sterol metabolism and oxidative stress response” by Edubiel et al., demonstrates that in laboratory generated AmB resistant cell lines, wild type ergosterol is replaced by other sterol intermediates. Further, WGS of these cell lines revealed mutations in sterol methyl transferase and/or sterol C5 desaturase. In some cell lines, additional deletion of miltefosine transporter gene was also observed. However, most of the resistant cell lines (10/14) did not exhibit virulence both under in vitro and in vivo conditions suggesting loss of virulence.

The study did not generate any new information regarding mechanism of AmB resistance in laboratory generated resistant cell lines of L. mexicana ( as per ref nos. 93-104) and other Leishmania spp. Present manuscript is mere repetition of the same with some more cell lines. To understand whether, same deduced mechanism of AmB resistance in laboratory is indeed prevail in field, the study has to be performed with AmB resistant clinical isolats taking laboratory resistant cells as control. These clinical isolates should also be characterized in terms of in vivo resistant phenotype, virulence and cross resistance. Identified genes can be evaluated for their expression levels. Further to understand mechanism of cross resistance, comparative sterol profile and differential expression of sterol biosynthetic pathway may also be studied in mitefosine resistant lines. Hence,manuscript in present form is not recommended for publication.
