# Supplementary material for: Amphotericin B resistance in Leishmania mexicana: Alterations to sterol metabolism and oxidative stress response
Source: PLoS Negl Trop Dis. 2022 Sep 28;16(9):e0010779. doi: 10.1371/journal.pntd.0010779 (PMC9581426; doi:10.1371/journal.pntd.0010779)
Supplement: Attachment — Submitted filename: Letter_of_response_to_Reviewers_comments.docx [file pntd.0010779.s023.docx]

**Point-to-point Response to the Reviewer’s Comments**

**Manuscript Number:** PNTD-D-22-00489 - [EMID:3adf66320527310e]

**Authors:** Alpizar-Sosa E., et al.

The authors thankfully acknowledge the recommendation of the Editors to revise the manuscript by addressing the comments suggested by the reviewers. The authors are also appreciating the reviewer’s positive feedback and constructive criticisms of this work, which overall contributes towards improving the quality of this manuscript.

The authors, hereby, submit the revised manuscript for re-review as a full-length article. The revised manuscript, along with the point-to-point response to the reviewer’s comments, is submitted accordingly. All changes in the revised manuscript are highlighted in “yellow” or in “~~red and strikethrough~~” in the case of deleted content). Additionally, text highlighted in “turquoise” indicates unmodified content (relative to the previous version submitted) where responses to some of the reviewers queries are found. This version also includes all the changes suggested by the Editorial Team (e.g. remove trademarks, etc) and other typos found along the process. Following the same rule, we have removed and highlighted those changes requested by the PlosNTD Editorial Team in both the corrected and clean versions of the manuscript.

As indicated in the manuscript, the genomics and metabolomics datasets have been uploaded to the **NCBI** and **Metabolights** repositories, however these cannot be accessed until publication data (Dec 31 2022 for **NCBI**) or are not of public (**Metabolights**). Therefore, we provide the temporary link for the former, and as advised by **Metabolights** supporting team, we provide a screenshot which shows that files have been uploaded but the project (MTBLS2744) is under VALIDATION and cannot be accessed (is not public) until has passed this process. Therefore, we have also included a screenshot of the email from **Metabolights** stating this.

**Response to the reviewers:**

**Response:** The authors would like to thank the reviewer for the valuable feedback which helped a great deal in enhancing the quality of the manuscript. Also, the authors gratefully acknowledge the suggestions/concerns regarding incorporation of additional information to emphasize the new contributions of the present study and, addressing the differences - a numbered list is provided - between the present and our previous work on AmB (Pountain et al, 2019. Likewise, we agree that discussing the role of both pentamidine and paromomycin as well as expanding on the importance of AmB resistance in *L. donovani* will benefit the impact of our findings. Additionally, we have also included information related with the use of AmB (and regardless of this been indicated for VL) in cases of CL Leishmaniasis, therefore the importance of this work with regard of the potential risk of AmB resistance and cross resistance with other antileishmanials.

**Reviewer #1:**

Alpizar-Sosa et al. are presenting a very detailed and comprehensive study of in vitro AmB in the parasite Leishmania. Overall, the work has been carried out expertly with many attentions to details. They are similarities with their previous study of Pountain et al., that was published in PNTD in 2019 but also a number of differences that bring new perspectives on the mode of action of AmB in Leishmania. Here for the first time, they present data of cells selected for resistance to nystatin. Interestingly they have shown differences in AmB activity depending on the medium and furthered these studies with state-of-the-art metabolomic work. Their collateral sensitivity to paromomycin (1), pentamidine (2) and others (3) is of interest and should also be emphasized (4). Since the genes discovered (C5DS, C24SMT, MT) are similar, and their surprising finding that resistant cells retained their virulence have already been reported in their 2019 paper I would suggest to the authors to emphasize how the current study distinguishes itself from the previous one.

**Comment 1:** I am not sure I understand their statement on line 146-7. Why is their WT not growing when passing from HOMEM to DM?; did they not select WT cells for resistance when grown in DM?

**Response:** The authors agree to the concern raised by the esteemed reviewer and do feel that the statement in its current form may arouse confusion. As described in section “Cell culture and polyene-resistant selection (page 31, line 655-666), we selected independent lines in two different culture media, namely HOMEM and serum free Defined Medium (DM). Formulation of both media is provided (S2 Table). To clarify, however, the following two sentences have been modified to highlight the fact that DM and HOMEM cell lines were maintained separately.

(Page 33, line 711-714):

*“L. mexicana cells were selected for resistance by stepwise increasing concentrations of AmB (ten lines) in either HOMEM (thirty-two weeks) starting from 50 nM and rising to 220-300 nM (S1 Fig-A and C) ~~and~~ or in serum-free defined medium DM (fifty weeks) from 100 nM up to 300 nM (S1 Fig-D).”*

(Page 33, line 719-721):

*“Retention of resistance (as promastigotes) of all independent lines was confirmed after at least ten passages in their respective culture media (HOMEM or DM) without drug pressure and post-infection in vivo in all resistant lines (n=5) and wild type included in these experiments.”*

Accordingly, the following sentence (and including S2D Fig) has been removed from the manuscript since we believe that adds no relevant information to our findings (note that former S2E Fig is now S2D Fig, line 152):

(Page 8, line 173-175):

*“~~Interestingly, four AmBR lines sustained in HOMEM alongside the parental line showed no growth after switching from HOMEM to DM (S2D Fig).~~”*.

(Page 50, line 1411):

“**~~D) Growth kinetics of AmBR lines from panel A (HOMEM) cultured in DM. C)~~D) Violin plot of the mean**”

**Comment 2:** In conclusion a very complete and comprehensive study but I encourage to highlight more clearly how this study distinguishes from their previous one (outside studying 15 lines instead of 4).

**Response:** The authors are thankful to the esteemed reviewer for this compliments and insightful review and pertinent suggestions. Accordingly, the authors have addressed and highlighted, modified, or added, the sentences listed below, to emphasize the new contributions of this study, in comparison with our previous works with AmB in *L. mexicana* (Mwenechanya et al., 2017 and Pountain et al., 2019). Briefly, the list of new findings includes seven groups listed (lines 501-518) and discussed below:

(1) **Selection for resistance to AmB in two different culture formulations:** Clearly, our present work used **(i)** HOMEM supplemented with fetal bovine serum (FBS) and **(ii)** in a serum and lipid free Defined Medium (DM). Previous studies of AmB resistance in *L. mexicana* and other *Leishmania* spp., including our two studies (Mwenechanya et al., 2017 and Pountain et al., 2019) have maintained and selected parasites under drug pressure using media supplemented with 10 to 20% of fetal bovine serum (FBS). To the best of our knowledge, the present is first study in any *Leishmania* species addressing the selection of drug resistance to AmB (and possibly to other antileishmanials) under serum free culture medium. Accordingly, the authors have emphasized the use of HOMEM and DM culture conditions across the manuscript in the following sentences:

(Page 17, line 357):

*“One group of three resistant lines cultured in HOMEM revealed point mutations to C5DS…”*

(Page 18, line 392-395):

*“Interestingly, this pre-existent variant was found in AmBR lines (including wild types) cultured in both DM and HOMEM media (Table 1) which can be explained since it constitutes a region that differs between the two copies of C24SMT and is associated with structural changes but not with resistance to AmB (94).”*

(Page 19, line 418-428):

“*Here we noted that in three lines cultured in HOMEM (AmBRcl.8, AmBRcl.6 and AmBRA4) the MT and an adjacent gene (LmxM.13.1540) were deleted (S7C-D Fig). In AmBRA4 the deleted region was larger (~20 kb) spanning two additional genes (S7D Fig). Moreover, in one line, AmBRC1, also cultured in HOMEM, a homozygous variant G1041A (W347*) appears (Fig 5C & Table 1), indicating a translation termination (stop codon) and consequent loss of function of MT that resulted in 3.4-fold resistance to MF relative to WT (Fig 1B & S3 Table). Further changes in ploidy were noted in four HOMEM lines. A reduction in CNV in the MT locus was also found in AmBRcl.14, AmBRcl.3 and NysRcl.B2 (they lost one copy of the two) but increased in AmBRB2. CNV analysis confirmed that the MT gene was, however, retained in all of the other lines selected in HOMEM. Notably, in all the lines selected in DM, the MT gene remains unaltered*”

(Page 20, line 434-444):

*“…found altered. Notably, none of these mutations are present in cell lines selected in DM but they appeared only in HOMEM-mutants ~~medium~~ in which oxidative stress was more profound (Fig 2). A further gene was found mutated in another HOMEM line, AmBRcl.G5 of ~~(~~L. infantum~~)~~, this novel variant a heterozygous non-synonymous mutation G575C (S192T) (S9A Fig) was found ~~present~~ in the glutathionylspermidine synthase (GSS) encoding gene (ID = LINF_250031200, formerly annotated as LinJ25.2500). Strikingly, the same residue G575C (S192T) in GSS (LmxM.25.2380) was also mutated in another two HOMEM grown L. mexicana lines, AmBRcl.8 and AmBRcl.6 (S9C Fig) both which showed an attenuated phenotype in mice (Fig 6-A) as ~~and~~ discussed in the following section ~~later~~. Moreover, in five HOMEM grown lines (AmBRcl.14 and AmBRcl.3, AmBRcl.8, AmBRcl.6 and NysRcl.B2), CNV revealed moderate changes (i.e. they lost one copy) in ~~another~~ four genes …”*

(Page 21, line 447-455):

*“Previous work has pointed that GSS catalyses the synthesis and degradation of glutathionylspermidine, which is involved in maintaining intracellular thiol redox and in defense against oxidants and the~~. to a~~ potential role of the leishmanial redox system (S9B Fig) in resistance to AmB (59,82,86–88,90,116,130,131). The actual role of these genes awaits further investigation to determine a mechanistic explanation and, as noted earlier, evidence on relative sensitivity indicates that polyene resistant lines selected for resistance to AmB in HOMEM, are notably ~~actually~~ more sensitive to various agents associated with oxidative stress (S3 Table), a feature also noted for AmBR Candida strains (76).”*

(2) **Selection in parallel for resistance to another polyene antifungal, nystatin**: To the best of our knowledge, has not been previously investigated in *Leishmania* spp. The authors believe that the fact that nystatin was investigated for resistance in *Leishmania* for the first time is clear and that extensive evidence and findings identified in the four Nystatin resistant mutants described here, is sufficient and highlighted across the entire manuscript. Some examples of these are shown below:

(Page 2, line 35, Abstract):

“…*resistance to either amphotericin B or the related polyene antimicrobial, nystatin.*”

(Page 32, line 715-716):

“*Nystatin was added to L. mexicana (four lines) in HOMEM from 1.5 µM - up to 12.5 µM (S1 Fig-C)*.”

(Page 17, line 357-360):

*“One group of three resistant lines cultured in HOMEM revealed point mutations to C5DS (or lathosterol oxidase (LOX), gene ID = LmxM.23.1300). Notably, we identified variants in cell lines resistant to both polyenes AmB and for the first time in* *Leishmania mexicana, to the polyene nystatin.”*

(3) **Use of Metabolomics (LC-MS) to determine the effects of exposure to AmB on the cellular metabolism of *Leishmania mexicana*.** At the present, the authors believe that no previous studies have addressed the analysis of the response of cellular metabolism of *Leishmania mexicana* resistant to AmB and in wild type after exposure to high concentrations of AmB (5 x the respective EC50 value of each line) for short time (15 minutes). This approach used here to analyze the metabolic response has been previously enabled the study of metabolic pathways (<https://doi.org/10.1017/S0031182010000077>) in response to other antiprotozoal drugs (DOI: 10.1371/journal.pntd.0001618). Accordingly, the authors added the following sentences (yellow) to emphasize previous content (turquoise) addressing the fact that the changes in metabolism identified are present in HOMEM, but not in DM cultured cells, as follows:

(Page 37, line 809-814):

“Mid-log L. mexicana promastigotes (1 x 10^8^) of all AmB-resistant lines alongside their parental wild-type were treated with AmB (Sigma®) at 5 x their EC_50_ for 15 mins as follows: the amount of AmB for lines selected in HOMEM was 0.3 μM for both parental lines (LmWT1 and LmWT2), 3 μM for AmBRcl.14 and AmBRcl.8, and 1.74 μM for AmBRB2. Both wild type (LmWT-DM) and AmBRA3-DM cultured in DM media were treated with AmB at 1.16 μM and 3.5 μM, respectively.”

(Page 30, line 627-650):

“In yeast, contradictory findings have been made regarding the role of oxidative stress. For example, enhanced oxidative stress resistance has also been implicated in loss of susceptibility to AmB in some studies (45,83,84), while increased sensitivity to oxidative stress was seen as a universal feature in Candida strains selected for AmBR (76). In Leishmania, other studies also pointed to oxidative stress response in AmBR (59,82,88,90,116,130,140). Here we show that the activity of AmB appears to be substantially augmented in serum and lipid rich medium (FCS supplemented HOMEM) and associated with enhanced induction of oxidative stress (including exposure at high concentrations for 15 mins) as judged by increased flux of glucose via the PPP in AmB treated cells. The activity of AmB against wild type L. mexicana grown in HOMEM containing FBS compared with serum-free DM showed the drug to be significantly more potent (3.6 to 4.11-fold) in the rich conditions (Fig 3A, B & D; S3 Table) which may point to enhanced oxidative stress causing this increased influx to regenerate NADPH lost in fuelling the redox defence pathways (116–118). Notably, too, beyond the loss of heterozygosity associated with C24SMT, parasites selected for AmB resistance in DM did not reveal additional genomic changes in any redox pathway genes as found in five AmBR lines (AmBRcl.14 and AmBRcl.3, AmBRcl.8, AmBRcl.6) and the nystatin resistant (NysRcl.B2), sustained in HOMEM, emphasising that environment-specific impacts play key roles in AmB mode of action and resistance. However, our data indicated that AmBR is associated with increases in sensitivity to oxidative stress, as previously reported for Candida (76) where possible compensatory changes enabling optimal growth after altering sterol metabolism may have occurred. Further probing of the combined changes to the system are needed to understand the relationship between parasites, sterol metabolism, oxidative stress and responses to AmB.”

(4) **Whole Genome sequencing (WGS)** and s**terol analyses (GC-MS):** In one of our previous studies (Pountain, et al, 2019) we described the mechanistic of structural changes in the sterol gene C24SMT, this study (alongside with that of Rastrojo et al, 2019 in *L. major* which was published at the same time) was the first to demonstrate and propose the mode of resistance at this locus and their role in structural genomics changes and resistance to AmB. In our present work, we found that the mutation G961A was present in various lines (Table 1) in this locus but noted a new fact previously unknown: while this variant is a pre-existing difference between the two copies of this gene associated with structural changes but not with drug resistance (therefore also found in some wild types), as we found previously, it is present as heterozygous in wild type cells but homozygous in AmBR cells cultured in serum free DM medium. Likewise, we here identified genomic changes in two other sterol or lipid genes, i.e. the sterol C5DS and the miltefosine transporter. In both cases, the present study is the first work to establish that these variants seem to be associated with the culture media conditions (mutations appeared only in those cell lines cultured in medium supplemented with serum [HOMEM] but not in any of the serum free [DM] resistant lines). Additionally, this is the first study with *L. mexicana* cells selected for resistance to polyenes other than AmB, i.e. nystatin and we described similar mutations in C5DS in these lines. Moreover, and unlike in any of our previous works with AmB, here we have also included one VL specie, *L. infantum*, that was selected for resistance to AmB in HOMEM. Accordingly, the authors added or modified the following sentences across the manuscript to emphasize the new contributions (and differentiate these from our previous work with *L. mexicana* AmBR lines), as follows:

(Page 17, line 357-360):

“One group of three resistant lines cultured in HOMEM revealed point mutations to C5DS (or lathosterol oxidase (LOX), gene ID = LmxM.23.1300). Notably, we identified variants in Leishmania mexicana cell lines resistant to both polyenes AmB and for the first time, to the polyene nystatin. In AmBRcl.3,...”

(Page 18, line 384-391):

“*Likewise, in all of the AmB resistant lines selected in DM, this intergenic region was not of decreased coverage (S6C Fig), but a homozygous variant G961A (V321I) was found. Wildtype cells were heterozygous for this variant (Table 1) thus a loss of heterozygosity in lines selected for AmBR in DM appears to have occurred. The accumulation of cholesta 5,7,22-trienol indicates that the duplicated allele is defective, leading to the pathway’s truncation at this point in the homozygous state. The mechanism is likely to be analogous to that described previously (94).*”

(Page 19, line 418-428):

“*Here we noted that in three lines cultured in HOMEM (AmBRcl.8, AmBRcl.6 and AmBRA4) the MT and an adjacent gene (LmxM.13.1540) were deleted (S7C-D Fig). In AmBRA4 the deleted region was larger (~20 kb) spanning two additional genes (S7D Fig). Moreover, in one line, AmBRC1, also cultured in HOMEM, a homozygous variant G1041A (W347*) appears (Fig 5C & Table 1), indicating a translation termination (stop codon) and consequent loss of function of MT that resulted in 3.4-fold resistance to MF relative to WT (Fig 1B & S3 Table). Further changes in ploidy were noted in four HOMEM lines. A reduction in CNV in the MT locus was also found in AmBRcl.14, AmBRcl.3 and NysRcl.B2 (they lost one copy of the two) but increased in AmBRB2. CNV analysis confirmed that the MT gene was, however, retained in all of the other lines selected in HOMEM. Notably, in all the lines selected in DM, the MT gene remains unaltered (S7E Fig)*.”

(Page 27-28, line 560-576):

“Such mutations have been discovered in laboratory-generated AmB-resistant parasites~~,~~ as well as clinical isolates~~,~~ of L. mexicana (93,94), L. donovani (59,95–97) and L. infantum (91)which were cultured in media supplemented with 10 to 20% foetal calf serum. The fact that L. donovani (59,95–97) and L. infantum (reported here) lines that have been selected for resistance to AmB share with L. mexicana mutations in sterol pathway enzymes supports the use of L. mexicana as a suitable model to discover mechanisms of resistance that are applicable to other leishmania species.

Apart from our previous work in which we analysed four AmBR lines (94), previous studies with AmB have focused on a few ~~individual single~~ mutants which ~~restricts method~~ has limited the ability to identify other ~~different~~ causes of resistance. Moreover, no previous worked has applied this method in serum and lipid free culture conditions. For this reason, here, we selected a total of 15 new mutants resistant to polyene antimicrobials including ten L. mexicana lines resistant to AmB (AmBR) (seven cultured in HOMEM medium containing FBS and three in a serum and lipid free DM) in the hope of expanding knowledge on mechanisms of resistance to AmB. A further four L. mexicana lines were selected in HOMEM for resistance to the polyene Nys (NysR), a relative of AmB and a single line of the VL causing, L. infantum, was selected for AmBR in HOMEM”

(Page 31, line 651-653):

” In all ~~of~~ the lines selected here in HOMEM, as well as many earlier selected lines (also cultured in the presence of serum), genes encoding enzymes involved in sterol metabolism are mutated.”

(Page 31, line 658-659):

“…architecture which are not present in serum free conditions that impact the interaction of AmB with the membrane…”

(Page 31, line 661-666):

“Here, we did not study the appearance of mutations across a time course, but it is possible that selection in low level AmB leads to changes in genes such as the MT, for example, in some lines before the acquisition of higher-level resistance through loss of drug binding sterols derived from mutations to genes involved in sterol synthesis. Earlier appearing mutations in the latter would preclude a need for mutations in the flippase since cells would have already exceeded the resistance level available through flippase-loss.”

(Page 31, line 671-679):

“However, such mutations are found in both L. mexicana and L. infantum so it is possible such changes might be required to enable changes to sterol metabolism when grown in rich medium as reported for Candida (76). Unlike the Candida study, however, leishmania selected in this way can retain virulence in mice. Two C5DS mutant lines retained their infectivity in mice. In these, two different independent mutations in the same residue (V74M) and two deletions (M93del and A96del) alongside the accumulation of ergosta-5,7,24-trienol, an ergostane-type sterol is associated with AmB resistance and virulence (55,94,126), were found. Previously a C24SMT associated mutant also retained virulence (94).

To further emphasize the contributions of the present work and in response to the **reviewer #1**, the authors thankfully acknowledge the relevance of this suggestion and therefore, here we provide additional changes made to emphasize our findings regarding the collateral sensitivity observed with the antileishmanials pentamidine and paromomycin. Furthermore, the importance of both antileishmanials have been included in the discussion, as advised by the editor:

(Page 32, line 680-699):

“The fact that AmBR has been rare in the field until now might simply be due to the fact that, until recently, it was not widely used, and has generally involved protracted dosing regimens. Extended use and the decision to use a single dose of the drug in monotherapy has great public health benefits (40,41,142,143) but that policy must consider the fact that selection of AmB resistance is clearly feasible in both CL and VL species, as we have shown here with L. mexicana (CL) and L. infantum (VL), and that resistance can be sustained in parasites that are virulent in mammals. Although CL is mainly treated with antileishmanials other than AmB, the Pan American Health Organisation has suggested its use in relapse cases for New World cutaneous species (171) although several reports indicate that liposomal AmB was unsatisfactory in treating CL caused by L. mexicana (172-174).

Another important point for consideration relates to the widespread administration of miltefosine in clinical populations which may carry a risk of cross resistance between AmB and MF (92,100,101,109) given that it seems loss of the miltefosine transporter can contribute to AmBR. Interestingly, here, as in other studies, AmB resistance is accompanied by increased sensitivity to the antileishmanials pentamidine and paromomycin. Data from L. major have suggested changes in mitochondrial membrane potential (102) related to lipid remodelling (111) might underlie pentamidine hypersensitivity, but mechanisms that could explain paromomycin hypersensitivity are not yet known. The possible use of pentamidine and paromomycin against AmBR lines should they become problematic in the field, however, deserves further consideration.”

The following references cited in the paragraph cited above which are related to the use of AmB in CL caused by *L. mexicana*, have been cited (1) and included to the list of references (2):

(Page 48, line 1316-1325):

171. Pan American Health Organization (PAHO). Leishmaniasis in the Americas: Treatment recommendations. Washington, D.C.: PAHO; 2018. ISBN: 978-92-75-11752-1. Available from:

[https://www.paho.org/en/documents/leishmaniasis-americas-recommendations-treatment-2018](https://www.paho.org/en/documents/leishmaniasis-americas-recommendations-treatment-2018 )

172. van Thiel PP, Zeegelaar JE, van Gool T, Faber WR, Kager PA. Cutaneous leishmaniasis in three Dutch military cohorts following jungle training in Belize. Travel Med Infect Dis. 2011;9(3):153-160.

173. Zeegelaar JE, Steketee WH, van Thiel PP, Wetsteyn JC, Kager PA, Faber WR. Changing pattern of imported cutaneous leishmaniasis in the Netherlands. Clin Exp Dermatol. 2005;30(1):1-5.

174. Eldin C, l'Ollivier C, Ranque S, Gautret P, Parola P. "Chiclero's Ulcer" Due to Leishmania mexicana in Travelers Returning from Central America: A Case Report and Review of the Literature. Pathogens. 2021;10(9):1112.

(5) **In vivo studies retention of resistance after infection in mice**. Unlike in our previous work, here we have used histological analysis of infected mice tissue, this allowed us to confirm the presence of parasites in animal tissue in addition to analyzing the retention of resistance after parasites were recovered from infected mice. This lead to identify an association between gene mutations and phenotype (i.e. sterol profile and infectivity) as well as confirming that in all lines, resistance to AmB was retained. Accordingly, the authors highlight here that only those HOMEM-selected (but none DM-selected) AmBR-lines in which variants were found in sterol genes were chosen for *in vivo* work, the following sentences were modified as follows:

(Page 23, line 503-508):

*“**We also tested whether the parasites from the four HOMEM lines with mutations in sterol genes and chosen* *for in vivo infection retained resistance to AmB within the mouse (Fig 6A) and ~~also~~ when recovered from primary lesions (footpad) (Fig 6B & S7 Table) and lymph nodes. After infection, primary lesion size was monitored weekly thereafter and mice were treated with AmB (six doses of 1 mg per kg given every other day, with higher doses being toxic to mice) at week eight of infection.”*

(Page 25, line 525-526):

“*Promastigote cultures of the four HOMEM AmBR lines recovered from footpads and lymph nodes of infected mice treated with AmB were established and EC50 values to AmB measured*”

(6) Additional to our replies to our esteem reviewers, here the authors listed the main findings from our previous study (Pountain et al, 2019), which were not pursued in the present work (therefore not discuss further), such as:
    a) RNA-seq of AmBR cell lines as promastigotes and amastigotes forms.
    b) Identification of SIDER1 elements in C24SMT locus highly homologous with the intergenic region of the sterol C-4 sterol methyl oxidase gene (LmxM.36.2540), resulting in multi-gene amplification and structural variants.
    c) Characterization, using (i) qPCR (ii) gel electrophoresis and (iii) Sanger sequencing, to determine first, the mechanistic of the chronological order of mutations between C24SMT and MT (this showed that changes in the C24SMT appeared by day 144 after the deletion of the miltefosine transporter (MT) by day 75-93 during drug selection) and second, the functional analysis of mutations found in genes C24SMT, MT and C5DS in add-back cell lines.
    d) Genotyping of the variants G391A and G961 to determine that these are not related with drug resistant but were pre-existent in the C24SMT locus (and are associated with structural variation).

**Comment 3:** BTW their statement on line 512 is not accurate as other published studies have studied more than one AmB resistant mutant.

**Response:**

The authors deeply acknowledge the thoughtful comment of the reviewer, and the authors recognize that the statement is not accurate and that other studies have addressed more than one resistant line. Accordingly, the authors have modified its content as shown below:

(Page 27, line 567-570):

*“Apart from our previous work in which we analysed four AmBR lines (94), previous studies with AmB have focused on a few ~~individual single~~ mutants which ~~restricts method~~ has limited the ability to identify other ~~different~~ causes of resistance. Moreover, no previous worked has applied this method in serum and lipid free culture conditions.”*

**Reviewer #2:**

The manuscript entitled “ Amphotericin resistance in L. mexicana : alteration to sterol metabolism and oxidative stress response” by Edubiel et al., demonstrates that in laboratory generated AmB resistant cell lines, wild type ergosterol is replaced by other sterol intermediates. Further, WGS of these cell lines revealed mutations in sterol methyl transferase and/or sterol C5 desaturase. In some cell lines, additional deletion of miltefosine transporter gene was also observed. However, most of the resistant cell lines (10/14) did not exhibit virulence both under in vitro and in vivo conditions suggesting loss of virulence.

The study did not generate any new information regarding mechanism of AmB resistance in laboratory generated resistant cell lines of L. mexicana (as per ref nos. 93-104) and other Leishmania spp. Present manuscript is mere repetition of the same with some more cell lines. To understand whether, same deduced mechanism of AmB resistance in laboratory is indeed prevail in field, the study has to be performed with AmB resistant clinical isolates taking laboratory resistant cells as control. These clinical isolates should also be characterized in terms of in vivo resistant phenotype, virulence and cross resistance. Identified genes can be evaluated for their expression levels. Further to understand mechanism of cross resistance, comparative sterol profile and differential expression of sterol biosynthetic pathway may also be studied in mitefosine resistant lines. Hence, manuscript in present form is not recommended for publication.

**Response:** The authors would like to thank the reviewer for the valuable feedback which helped a great deal in enhancing the quality of the present manuscript. Also, the authors gratefully acknowledge the suggestions/concerns regarding incorporation of new data from clinical isolates as well as further experiments to validate the deduced mode of action described here in polyene (AmB and Nystatin) resistant laboratory strains and our new findings shown in our present work. However, it is important to clarify that additional experimental validation in (1) *L. donovani* or any other VL species is tantamount to carrying out an (2) animal study at the least, if not a (3) clinical trial, which is going to be each a project in itself. Moreover, at the present testing *L. donovani* is not possible for us considering that *L. donovani* (and the same applies to any other VL species) are “human pathogen hazard group” which would require CAT3 facilities at least within the UK -approved List with effect from 12 July 2021- (<https://www.hse.gov.uk/pubns/misc208.pdf>) to which, unfortunately, the authors have no current access. Nevertheless, the authors unequivocally agree that pursuing investigating these findings in other VL species and including clinical isolates, is of the utmost importance should other researchers working in the field and with access to VL clinical samples have interest, the authors are happy and currently looking into collaborations to address this relevant point which very much would enhance our understanding on AmB resistance. It may also be noted that, using *Leishmania mexicana* (and any other CAT-2 species) is indeed important and very convenient model, as much as it is the ease of maintaining axenic promastigotes instead of amastigotes, given the readiness for maintaining the former in laboratory conditions. Hence, carrying out an animal study or a selection of clinical isolates would require additional experimentation and resources in the range of months. While the authors agree of the importance if this, however, clinical isolates (like any other laboratory strain was initially), will take considerably time to adapt into the ‘new’ laboratory culture conditions, and sometimes, this is not achieved and if so, cultured adapted cells will also display new mutations derived from the currently well know genome plasticity of these parasites, carrying out such studies presents new challenges.

Understanding the criticality of the above insight as reflected by the esteemed reviewer, the authors further decided to add the following sentence in the manuscript to highlight the pertinent limitation of the directly translate these findings onto VL species, data on *L. donovani* resistant to AmB is incorporated in the body of manuscript, including the discussion (as indicated by the editor), as shown below:

(7) **Selection in parallel for resistance to AmB in another specie, the VL causing *L. infantum* (alongside the CL- causing *L. mexicana*):**

(Page 5, line 116-121):

*“Although AmB is used preferentially against visceral leishmaniasis caused by L. donovani or L. infantum, rather than cutaneous leishmaniasis caused by L. mexicana, the high degree of similarity between leishmania species often enables observations in once organism to allow inference in others. The relative simplicity of working with L. mexicana makes it a popular model for obtaining knowledge that might apply to other Leishmania species more widely.”*

(Page 29, line 619-622):

“Since AmB resistance mechanisms often appear to be similar between L. donovani and L. mexicana (57, 82, 87-89) the risk of AmB resistance in visceral leishmaniasis must also be considered as real.”

The following paragraph is now added/modified to expand on the previous statement on some of the similarities often observed across species, importantly, here we emphasize that these similarities regarding the mode of resistance to AmB, has been described in *Leishmania,* as shown below:

(Page 27, line 563-566):

“The fact that L. donovani (59,95–97) and L. infantum (reported here) lines that have been selected for resistance to AmB share with L. mexicana mutations in sterol pathway enzymes supports the use of L. mexicana as a suitable model to discover mechanisms of resistance that are applicable to other leishmania species.”

In order to further substantiate our results, the authors first highlighted that while we did not examine *L. donovani*, in fact, we analysed one VL causing specie (*L. infantum*) in our current study, precisely because we pursued to identify some similarities across species. This, followed by emphasizing the mutations found in the redox-involved gene GSS in the AmBR resistant line of the VL causing specie, *L. infantum* and the striking similarities of these variants with those found in *L. mexicana*, this finding is to the best of our knowledge, reported for the first time in two different *Leishmania* species (one CL- and another VL-causing) selected in parallel under similar culture conditions, followed by addressing that the derived susceptibility to oxidative stress found here, has also been described in fungal species (i.e. *Candida* spp.)*,* as shown below:

(Page 28, line 574-576):

“*A further four L. mexicana lines were selected in HOMEM for resistance to the polyene Nys (NysR), a relative of AmB and a single line of the VL causing, L. infantum, was selected for AmBR in HOMEM.*”

(Page 20, line 436-442):

*“A further gene was found mutated in another HOMEM line, AmBRcl.G5 of ~~(~~L. infantum~~)~~, this novel variant a heterozygous non-synonymous mutation G575C (S192T) (S9A Fig) was found ~~present~~ in the glutathionylspermidine synthase (GSS) encoding gene (ID = LINF_250031200, formerly annotated as LinJ25.2500). Strikingly, the same residue G575C (S192T) in GSS (LmxM.25.2380) was also mutated in another two HOMEM grown L. mexicana lines, AmBRcl.8 and AmBRcl.6 (S9C Fig) both which showed an attenuated phenotype in mice (Fig 6-A) as ~~and~~ discussed in the following section ~~later~~.”*

(Page 24, line 499-501):

*“It is noteworthy that in Candida selected for AmBR in vitro changes to sterol composition were associated with resistance, but the phenotype was also associated with enhanced susceptibility to oxidative stress and universal loss of virulence in that study (76).”*

Following our interest to cover some of the comments from our esteem reviewer (“most of the resistant cell lines (10/14) did not exhibit virulence both under in vitro and in vivo conditions suggesting loss of virulence”), below we highlight some points where information clarifies first, that we chose only four HOMEM lines to be tested in mice, therefore, concluding that 10 lines were not virulent is somewhat inaccurate. Likewise, to analyse the retention of resistance in mice and after infection *in vivo*, here only the same four lines chosen for *in vivo* work, were studied after these were recovered from mice tissue. Again, the authors cannot assume that 10 lines were in virulent intra-macrophages (i.e. *in vitro*), since only four AmBR lines were tested. Note that in these studies, we also included the NysR line, as described in the manuscript. This has been previously addressed here (see the in vivo rebuttal in **lines 322 to 331 of this document**) and comments below:

(Page 23, line 467-470):

*“**Therefore, we chose four of the HOMEM AmBR mutants to test their ability to retain infectivity and virulence in mice. Two of these were from the C5DS mutant group (AmBRcl.14 and AmBRcl.3) and two from the C24SMT mutant group (AmBRcl.8 and AmBRcl.6).”*

(Page 29, line 602-622):

“*This indicates the potential of C24SMT mutants to propagate as AmBR cells in patients. L. major C24SMT-KO mutants derived by Mukherjee et al. (103) were of reduced virulence in mice, as were the two L. mexicana lines AmBRcl.8 and AmBRcl.6 with C24SMT mutations we tested here. However, Pountain et al. (94) earlier found a C24SMT mutant that retained virulence in mice. This indicates that it might be mutations other than those to C24SMT per se that are responsible for attenuation in mice. Here, for example, we identify changes to the glutathionylspermidine synthase (GSS) gene in both attenuated L. mexicana lines (AmBRcl.8 and AmBRcl.6) and in L. infantum (AmBRcl.G5) (S9A Fig), although whether these changes are associated with attenuation is not yet known and requires further analyses. While some lines had lost virulence, two separate lines selected here (AmBRcl.14 and AmBRcl.3) with variants in C5DS, both retained virulence and resistance to AmB in mice. In L. major C5DS-KO lines displayed only a small loss of virulence in mice (94,104), thus C5DS mutations can also be selected, yielding parasites that are both AmBR and still able to infect mammals. Earlier work in Candida (76) had noted loss of virulence associated with selection of AmBR as a universal feature. In Leishmania, however, the risk of selection of AmBR in Leishmania appears to be a genuine risk, albeit the selective pressures on amastigotes in treated patients being distinct from those applied to selecting resistance in cultured promastigotes. Since AmB resistance mechanisms often appear to be similar between L. donovani and L. mexicana (57, 82, 87-89) the risk of AmB resistance in visceral leishmaniasis must also be considered as real.*”

Whereas the present study demonstrates some similarities with our previous studies we strongly disagree with the comment from the esteem reviewer on the fact that the present study “did not generate any new information regarding mechanism of AmB resistance in laboratory generated resistant cell lines of L. mexicana (as per ref nos. 93-104) and other Leishmania spp. Present manuscript is mere repetition of the same with some more cell lines.”. As discussed, point-to-point along this document, there are substantial differences in the approaches employed here and more importantly, outstanding novel findings that the authors believe are of relevance for the understanding on the mode of action and resistance of AmB in *Leishmania* spp. Briefly, these new approaches are listed below and were discussed in detail alongside this document:

(1) **Selection for resistance to AmB in two different culture formulations.** (line 74 in this document)
(2) **Selection in parallel for resistance to another polyene antifungal, nystatin** (line 124 in this document)
(3) **Use of Metabolomics (LC-MS) to determine the effects of exposure to AmB (to high concentrations of drug) on the cellular metabolism of *Leishmania mexicana*** (line 141 in this document)
(4) **Whole Genome sequencing (WGS)** and s**terol analyses (GC-MS):** While the approach is similar, bioinformatic tools were different than in previous work, and importantly, the number of cell lines analysed rendered the identification of novel genes in multiple *Leishmania* species, associated with selection for resistance to polyenes AmB and Nys (see line 182 onwards in this document for a detailed description).
(5) **In vivo studies retention of resistance after infection in mice.** (line 312 in this document)
(6) **List of approaches not used here in comparison with our previous work** (see line 331 in this document)
(7) **Selection in parallel for resistance to AmB in another specie, the VL causing *L. infantum* (alongside the CL- causing *L. mexicana*)** (see line 403 in this document)

Our present study showed, as extensively described in this document, new findings that were identified. It may, however, be noted that the authors are aware of some similarities in the variants arose in some genes, however, SNPs were not the same and notably, some valuable **additional** information was found in these variants. The best example of this is the loss of heterozygosity in the variant *G961A (V321I)* (see line 210-216 in this document) which was found homozygous in some AmBR lines selected in DM. This further illustrates that the analysis of multiple lines, as we aimed initially, enhances the identification of new aspects previously unknown, as well as facilitating the probability of identifying **new** genes involved, as found here with GSS, being this another of the most important contributions of this study, in particular the fact that GSS showed an identical variant in both *L. mexicana* and *L. infantum*, which cause CL and VL, respectively. The following sentences are now added to further support this:

(Page 6, line 141-145):

*“. Since studies so far have indicated that mutations to different genes in the sterol pathway can lead to AmBR (93,94), and other instances of resistance showed no apparent change to sterol metabolism (86) we anticipate that by expanding the numbers of resistant lines selected could amplify our knowledge of the different mechanisms capable of yielding AmBR.”*

(Page 10, line 225-228):

“It has been reported that a range of Candida species in which AmBR arose through changes to sterol metabolism had increased in sensitivity to oxidative stress (76), and the data here point to similar associations in Leishmania.”

(Page 12-13, line 269-276):

“This observation may also explain why the fold-change in resistance of the lines selected in HOMEM (5.29 to 12.79-fold) is greater than those achieved in DM (1.36 to 2.54-fold) (Fig 1), as alterations in specific parts of oxidative stress response could enhance HOMEM-based resistance, although cross-resistance to MB is not seen in AmBR cells, indicating that any such response is nuanced, and the data actually indicate that in the lines selected here, decreased sensitivity to oxidative stress is not evident, indeed, as reported in Candida spp. (76) resistant lines showed increased sensitivity to oxidative stress.”

(Page 28, line 584-597):

“These observations are consistent with previous studies where C24SMT (59,94,96,97,95) and C5DS (94) have been found associated with development of AmBR. Moreover, experiments in L. major where C24SMT (103) and C5DS (104) genes were knocked out of WT parasites also showed that viable parasites with resistance to AmB could be recovered. In these two knockouts, however, the effects on other parts of the genome ~~was~~were not determined. In spite of the fact that our findings on fundamental changes to sterol metabolism genes have not found mutations in genes other that C24SMT and C5DS, a number of noteworthy novel findings have been made. For example, we demonstrate that AmB appears to induce oxidative stress in Leishmania, as inferred from the large increase of flux through the pentose phosphate pathway. This effect, however, is confined to parasites grown in rich medium with serum added and not in serum free defined medium. In addition, parasites selected for resistance in rich medium including serum reveal multiple mutations in addition to those in the sterol pathway genes, while in DM these additional mutations are not evident.”
